# Anesthetic Management of Suspected COVID-19 Vaccination Pericarditis/Myocarditis Scheduled for a Pericardial Window: A Case Report and Literature Review

**DOI:** 10.7759/cureus.39222

**Published:** 2023-05-19

**Authors:** Cecilia Karemingi, Kateryna Georgiyeva, Elizabeth Santiesteban, John Sciarra, Harendra Kumar

**Affiliations:** 1 Anesthesiology, Larkin Community Hospital, South Miami, USA; 2 Anesthesiology, University of Rochester, Rochester, USA; 3 Internal Medicine, Memorial Healthcare System, Pembroke Pines, USA; 4 Anesthesiology, Larkin Community Hospital, South Miami, USA; 5 Medicine and Surgery, Dow University of Health Sciences, Karachi, PAK

**Keywords:** intra operative arrhythmia, anesthetic management, pericardial window, echocardiography - pericardial - effusion, transesophageal echocardiography (tee), covid vaccine-induced myocarditis, covid pericarditis, covid-19 vaccination

## Abstract

The unique challenges posed by the COVID vaccination continue to affect multiple healthcare specialties. Although short-term studies have shown that COVID-19 vaccines are both safe and effective, reports of side effects continue to emerge. Cardiovascular side effects such as myo-pericardial inflammation are of particular interest to the fields of cardiology, anesthesiology, and surgery. Myocarditis and pericarditis necessitate diagnostic and therapeutic procedures such as transesophageal echocardiography (TEE) and pericardial window surgery. Intraoperative monitoring of clinical status and heart rhythm and careful adjustments to anesthetic management are required to ensure successful outcomes.

This case report follows a 50-year-old male with a known history of pericardial effusion post-COVID vaccination who presented to the emergency department with shortness of breath and chest pain, necessitating further management. We examine the importance of TEE in preventing unnecessary pericardial window procedures and shed light on the importance of careful patient monitoring and management in promoting successful outcomes from an anesthesiology perspective.

## Introduction

Pericarditis and myocarditis are inflammatory diseases affecting the sac surrounding the heart and the heart muscle, respectively. Inflammation of this nature may cause chest pain, shortness of breath, and, in severe cases, heart failure. While the causes of these illnesses are often unknown, current research has identified a possible link between COVID-19 vaccination and the development of pericarditis and myocarditis [1].

Pericarditis and myocarditis primarily affect middle-aged men, with a yearly incidence of 1-10 cases per 100,000 people. The overall incidence of these disorders is difficult to estimate since many cases go untreated or present with minor symptoms. However, pericarditis and myocarditis may cause significant morbidity and mortality, particularly in cases with substantial cardiac involvement [1,2].

Recent research has shown a link between COVID-19 vaccination, particularly mRNA-based vaccines, and the development of pericarditis and myocarditis [1,2]. The frequency of these disorders after immunization is estimated to be one to five cases per 100,000 people, with young and middle-aged males bearing the most significant risk [2]. While the risk of increased morbidity and mortality in these situations is still being investigated, there have been reports of severe cases requiring hospitalization and supportive care [2,3].

Furthermore, the risk of myocardial instability during anesthetic treatment has been documented in patients with pericarditis and myocarditis, particularly in cases with underlying cardiac rhythm abnormalities. This presents a significant challenge for anesthesiologists caring for patients undergoing pericardial window surgery, which may need general anesthesia [4].

The anesthetic management of a patient with possible COVID-19 vaccination-related pericarditis or myocarditis who developed a substantial pericardial effusion is described in this case report. We emphasize the need for meticulous anesthetic preparation and a multidisciplinary approach to managing individuals who may have vaccine-related cardiac issues. The case also sheds light on the potential risks associated with the COVID-19 vaccination and the need for ongoing monitoring and investigation [2,4].

## Case presentation

A 50-year-old incarcerated male with multiple comorbidities, including a history of recurrent pericardial effusion, hypertension, type 2 diabetes with neuropathy, hyperlipidemia, obesity, and sleep apnea, presented to the emergency department with complaints of shortness of breath, positional chest pain, and symptoms of a flu-like illness. Notably, he had a history of two separate cases of pericardial effusion 1.5 and 2 years prior, presenting with identical symptoms. These cases coincided precisely with the time period during which the patient received his first dose of the Pfizer-BioNTech COVID-19 vaccine on May 19, 2021. Booster doses were administered on December 9, 2021, and February 22, 2023.

The patient received codeine-guaifenesin 10-100 mg, methylprednisolone IVP 80 mg, azithromycin 500 mg IV, and furosemide 40 mg IV in the emergency department and was admitted to the internal medicine floor for observation.

On admission, his vital signs were HR 90, BP 134/78, SPO2 100%, and RR 15. His laboratory workup showed an erythrocyte sedimentation rate (ESR) greater than 100 mm/hr. His height and weight were 156 cm and 156 kg, respectively. On examination, he was found to be morbidly obese and fully alert and oriented. His head was normocephalic and atraumatic, with reactive pupils. He had no nasal discharge, and moist mucous membranes were noted in the throat. The neck was supple and non-tender, without lymphadenopathy, masses, or thyromegaly, and there was no jugular venous distention (JVD) or carotid bruits bilaterally. On cardiac examination, S1 and S2 were faint, with no murmurs/rubs/gallops, no S3, no S4, and a regular rhythm and rate.

Peripheral edema was noted, with no JVD. Decreased breath sounds were heard at the base of the lungs with wheezing. The abdominal and musculoskeletal system assessments were unremarkable. Peripheral pulses were intact and 2+ bilaterally. Examination of both feet revealed all toes to be expected in size and symmetry, with a normal range of motion and normal sensation with a distal capillary filling of fewer than two seconds without tenderness, swelling, discoloration, nodules, weakness, or deformity. His skin was dry and undamaged, showing no signs of bruising or ecchymosis. Pertinent laboratory results are noted in Tables 1-3.

**Table 1:** Hematology laboratory results.

Test	Result	Reference range
White blood cell count	9.99	4.5 to 11.0 × 10^3^/μL
Red blood cell count	3.84	4.5 to 5.5 × 10^6^/μL
Hemoglobin	10.2	13.5 to 17.5 g/dL
Hematocrit	32.7	38.8–50.0%
Mean corpuscular volume	85.2	80.0–96.0 fL
Mean corpuscular hemoglobin	26.6	27.0–32.0 pg
Mean corpuscular hemoglobin concentration	31.2	32.0–36.0 g/dL
Red cell distribution width	14	11.5–14.5%
Red cell distribution width-standard deviation	43.7	37.0–54.0 fL
Platelets	517	150 to 450 × 10^3^/μL
Mean platelet volume	9.5	7.5–11.5 fL
Percentage of neutrophils	54.9	40.0–75.0%
Percentage of lymphocytes	33.7	20.0–45.0%
Percentage of monocytes	8.7	0.0–8.0%
Percentage of eosinophils	1.8	0.0–5.0%
Percentage of basophils	0.6	0.0–2.0%
Absolute neutrophil count	5.48	2.0 to 7.5 × 10^3^/μL
Absolute lymphocyte count	3.37	1.0 to 4.8 × 10^3^/μL
Absolute monocyte count	0.87	0.0 to 0.8 × 10^3^/μL
Absolute eosinophil count	0.18	0.0 to 0.4 × 10^3^/μL
Absolute basophil count	0.06	0.0 to 0.2 × 10^3^/μL

**Table 2:** Table of chemistry lab results with reference range.

Test	Result	Reference range
Glucose	111	70–100 mg/dL
Blood urea nitrogen	22	7–20 mg/dL
Sodium	136	135–145 mmol/L
Potassium	4.1	3.5–5.0 mmol/L
Chloride	97	98–107 mmol/L
Carbon dioxide	32	22–29 mmol/L
Calcium	8.8	8.6–10.3 mg/dL
Total protein	6.8	6.0–8.0 g/dL
Albumin	3.4	3.5–5.0 g/dL
Bilirubin	0.3	0.0–1.2 mg/dL
Aspartate aminotransferase	29	0–40 U/L
Alanine aminotransferase	36	0–44 U/L
Alkaline phosphatase	75	40–129 U/L
Blood urea nitrogen/creatinine ratio	25	20-Oct
Osmolality	276	275–295 mOsm/kg
Globulin	3.4	2.2–4.2 g/dL
Albumin/globulin ratio	1	1.2–2.2
Magnesium	2	1.7–2.6 mg/dL
Phosphorus	4.4	2.5–4.5 mg/dL
Glomerular filtration rate	97	≥60 mL/min/1.73 m²

**Table 3:** Special chemistry, microbiology, and urinalysis test results.

Test name	Result	Reference range
Troponin	<0.012	<0.040 ng/mL
Brain-type natriuretic peptide	410 (H)	<125 pg/mL
COVID	Negative	Negative
Procalcitonin	0.068	<0.050 ng/mL
Thyroid-stimulating hormone	1.33	0.40–4.50 µIU/mL
Urinalysis	Result	Reference range
Color	Yellow	Yellow
Appearance	Clear	Clear
Specific gravity	1.015	1.005–1.030
Urine pH	7	4.5–8.0
Nitrite, glucose, ketone, protein, blood, bilirubin	Negative	Negative
Urobilinogen	0.2	<2.0 mg/dL
Leukocyte esterase	Negative	Negative
Urinalysis microscopy	None seen	None seen

**Figure 1:**
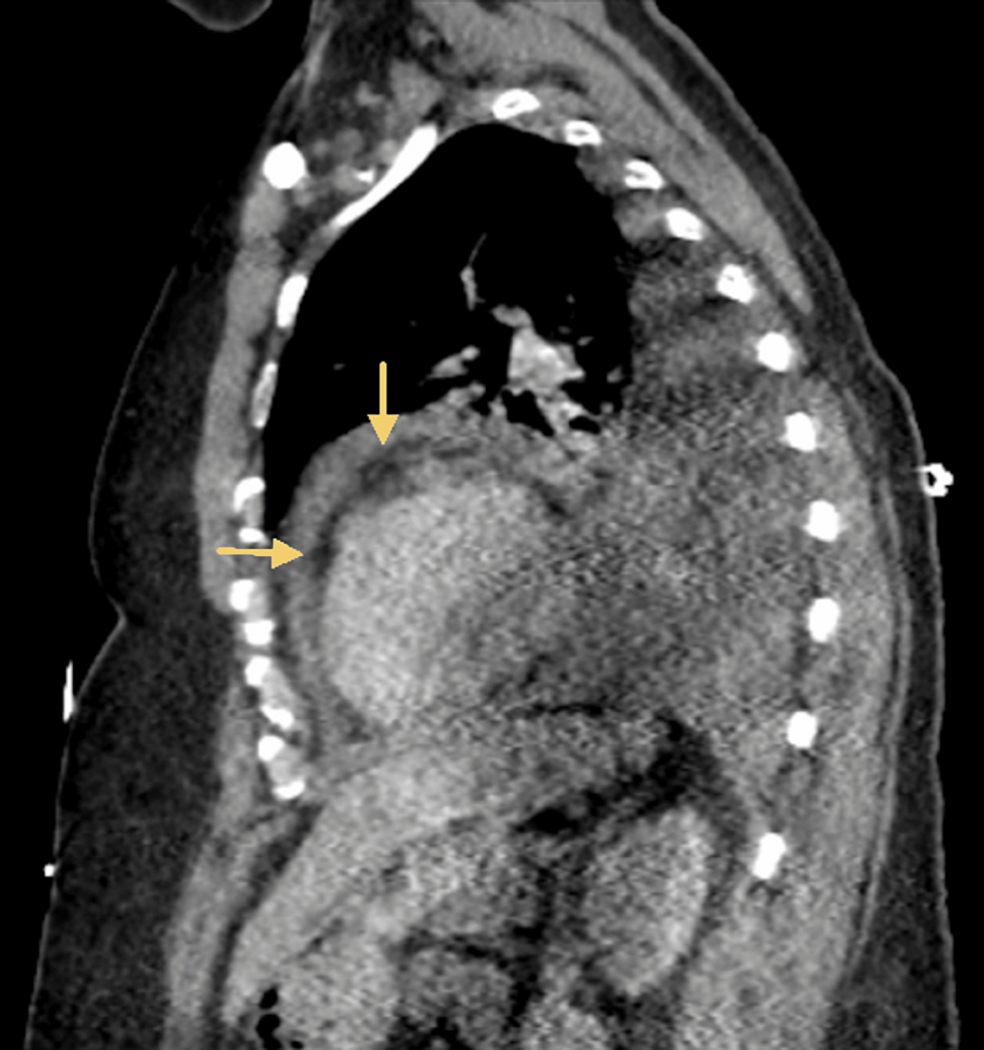
Computed tomography sagittal view of thoracic cavity. Note small pericardial effusion surrounding the myocardium (arrows).

**Figure 2:**
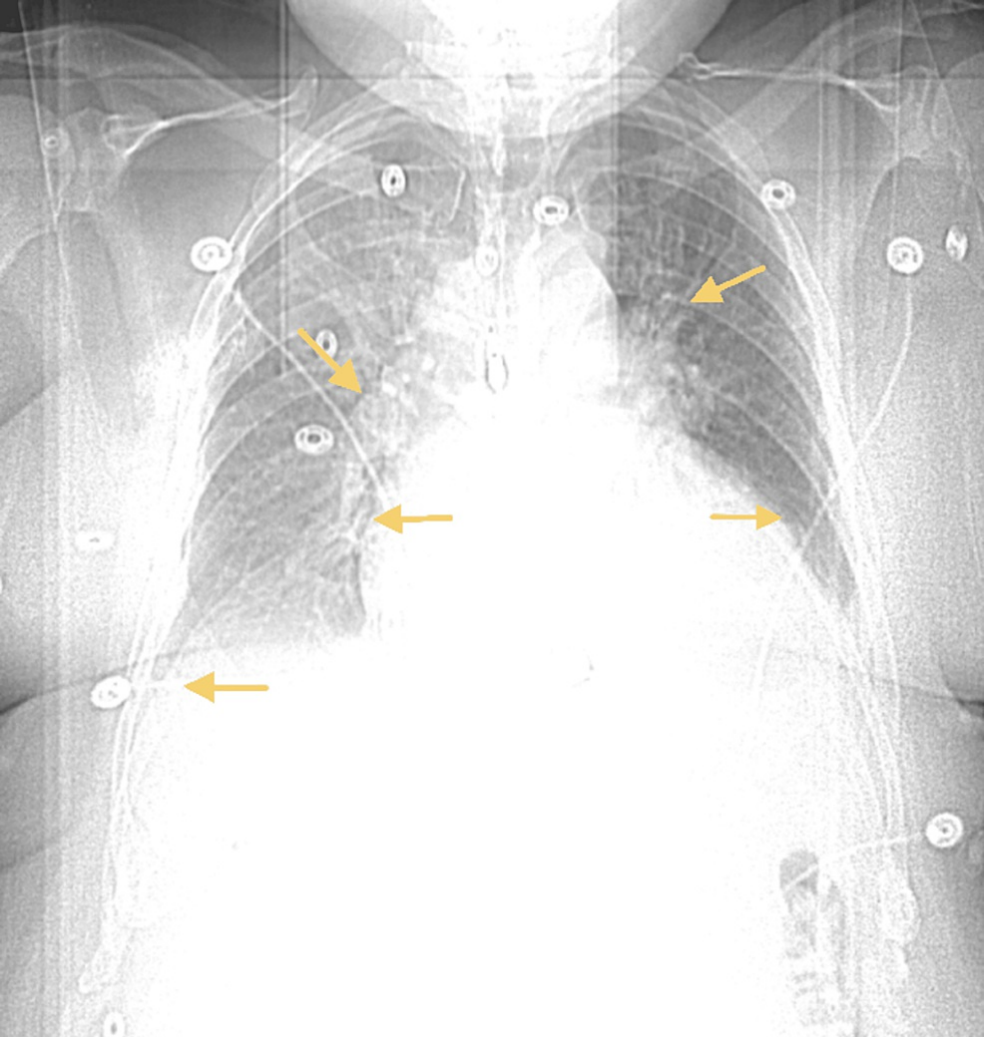
Chest radiograph coronal view. (a) Moderate volume bilateral pleural effusions with overlying atelectasis (superior arrows). (b) Rounding and enlargement of myocardium, evidencing inflammation, and accumulation of pericardial fluid (medial arrows). (c) Blunting of costophrenic angles (inferior arrow).

A chest X-ray revealed bilateral lower lung opacities. As shown in Figure 1, a computed tomography (CT) scan of the thoracic cavity in sagittal view demonstrated a small pericardial effusion surrounding the myocardium. Figure 2 shows a chest radiograph of the thoracic cavity in a coronal view, demonstrating moderate volume bilateral pleural effusions with overlying atelectasis, rounding and enlargement of the myocardial tissue due to inflammatory pericardial fluid, and blunting of the costophrenic angles. A transthoracic echocardiogram (TTE) was also performed, which showed a left ventricular ejection fraction of 65-70% with trace mitral and tricuspid regurgitation, pericardial effusion, and mild to moderate concentric left ventricular hypertrophy. The patient reported experiencing similar symptoms two years ago at the time of COVID vaccination, had an echocardiogram done, and was treated for pericardial effusion at that time, although he did not recall the specifics. The patient was started on anti-inflammatory medications (indomethacin and colchicine) for the pericardial effusion, and a pericardial window was scheduled five days later.

The patient arrived in the preoperative anesthesia holding area, where his vital signs were checked and procedure details were discussed. The patient had a Mallampati class III airway, good mouth opening and neck range of motion, and a thyromental distance of over 7 cm. A peripherally inserted central catheter (PICC) line was placed in the right brachial vein, and 1 mg of fentanyl intravenously (IV) was given to calm and sedate him. The patient was then taken to the operating room for the pericardial window procedure. Before induction, an arterial line was placed in the left radial artery for intraoperative monitoring. General anesthesia was inducted with etomidate, ketamine, and rocuronium.

**Figure 3:**
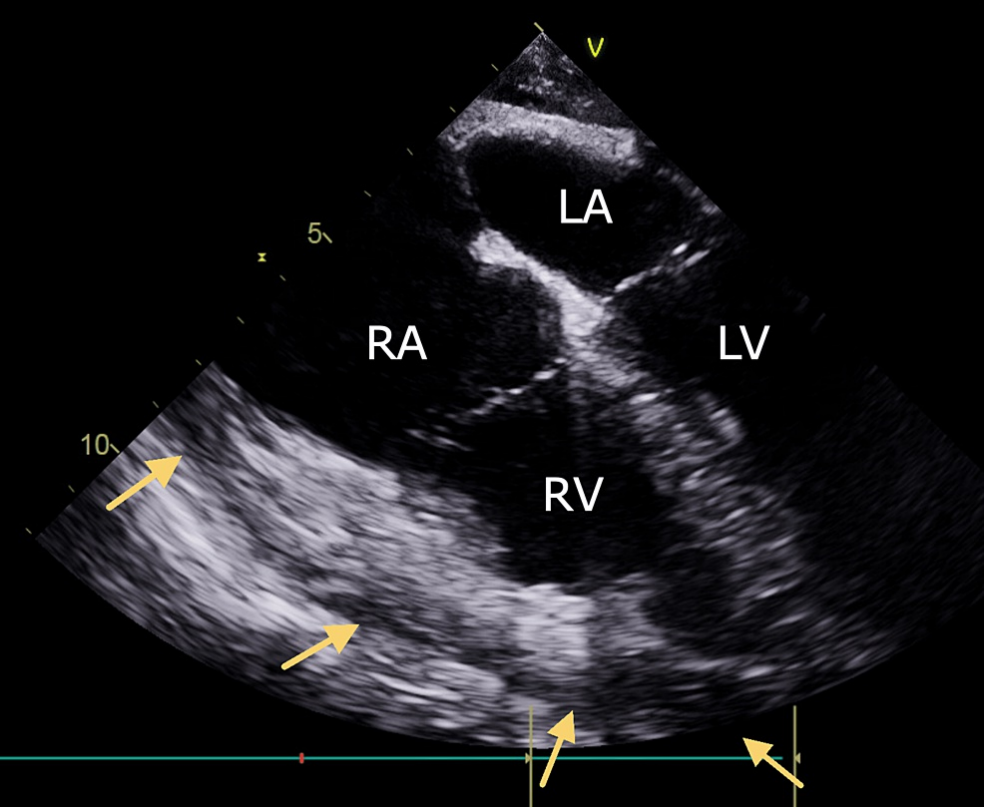
Intraoperative transesophageal cardiac echocardiogram four chamber heart view. Note small pericardial effusion surrounding the myocardium (arrows). RA: right atrium; LA: left atrium; RV: right ventricle; LV: left ventricle.

Prior to the surgical incision, a transesophageal echo probe was placed, and a pre-operative study was done. The chief anesthesiologist performing the echocardiography did not see any effusion of note, and the cardiothoracic surgeon confirmed this with cardiology. Therefore, the pericardial window procedure was deemed unnecessary by all teams involved. As shown in Figure 3, transesophageal echocardiography (TEE) prior to the planned pericardial window avoided the need for a pericardial window procedure that is not standard practice at all institutions.

After deeming the pericardial window unnecessary, the patient was given 200 mg of sugammadex to reverse the effects of the rocuronium used during anesthesia induction. However, a minute after receiving the sugammadex, the patient's heart rate became bradycardic at 40 bpm, which was corrected with atropine. Shortly after that, the heart rate spiked to 180 bpm, likely in response to the atropine. Sevoflurane inhalation anesthesia was terminated, and the patient progressed through stage 2 of consciousness and was successfully extubated. After the patient was fully awake, he was transferred to the post-anesthesia care unit. Atrial fibrillation was noted, and IV propranolol was administered to convert the patient's heart rhythm to a normal sinus rhythm. The patient was then transferred to the intensive care unit for further monitoring and was downgraded back to the internal medicine floor within 24 hours.

The patient was discharged with continued treatment for pericardial effusion: indomethacin and colchicine. Cardiac magnetic resonance imaging (MRI) was recommended as an outpatient procedure as well as for long-term follow-up with cardiology. A continuous positive airway pressure (CPAP) machine and neurology follow-up for obstructive sleep disorder were also recommended.

## Discussion

The exact mechanism of pericarditis and myocarditis after COVID-19 vaccination remains unclear. However, it is hypothesized that the immune response to the vaccine may cause an inflammatory response in the heart tissue, resulting in these issues [1,2,4]. The mRNA-based COVID-19 immunizations, in particular, have been associated with developing pericarditis and myocarditis. Studies indicate that the vaccine may cause an overly aggressive immune response, resulting in inflammation in the heart tissue [1]. The risk of pericarditis and myocarditis after COVID-19 vaccination is estimated to be one to five cases per 100,000 people, with a higher risk in young and middle-aged males [2]. However, the exact prevalence of these disorders in the vaccinated population is challenging since many cases are misdiagnosed or appear with minimal symptoms. While the risk of increased morbidity and mortality from pericarditis and myocarditis after COVID-19 vaccination is still being studied, there have been reports of severe cases requiring hospitalization and supportive care [4]. The danger of repercussions is exceptionally high in cases of considerable heart involvement, such as cardiac tamponade or sudden heart failure [3,4].

Following COVID-19 vaccination, patients may exhibit clinical symptoms and signs of pericarditis and myocarditis, including chest pain, shortness of breath, fever, and malaise. Elevated levels of cardiac enzymes such as troponin and creatine kinase-MB may be detected through laboratory tests. At the same time, TTE may reveal pericardial effusion, decreased ejection fraction, and abnormalities in regional wall motion. A TEE may also be necessary to screen for tamponade physiology and guide surgery [3,5,6].

In cases of pericarditis, a procedure known as a pericardial window may be necessary to drain excess fluid accumulated around the heart. However, there are risks associated with this procedure, including the likelihood of heart function impairment due to the anesthesia used [2,4,5]. As a result, it is recommended that a TEE be performed before any pericardial window procedure to establish if the fluid accumulation is severe enough to warrant intervention [1,4].

It is standard practice at some, but not all, institutions to perform TEE for pericardial effusions. Still, it is recommended in cases like this to prevent unnecessary interventions like a pericardial window and improve surgical outcomes. Due to the morbidly obese body habitus of our patient, a transesophageal echocardiogram was chosen instead of a transthoracic echocardiogram for better pericardial effusion visualization before the pericardial window. Patients with COVID-19-associated peri/myocarditis should be monitored frequently after treatment, as the condition may quickly resolve. Abnormally increased myocardial sensitivity in COVID-19 can cause a variety of disturbed rhythms, as seen in this patient: supraventricular tachycardia (SVT), sinus bradycardia, and atrial fibrillation [5]. Another important potential cause of bradycardia in patients with cardiac issues is Sugammadex, a modified gamma-cyclodextrin that is used to reverse steroidal non-depolarizing neuromuscular blocking drugs [5,6].

As a result, continuous monitoring of vital signs and imaging is essential to detect any changes in the patient's condition and give appropriate treatment. Furthermore, ongoing monitoring of the patient's vital signs and imaging studies is required to detect any changes in their health. With good medical care, improvements may occur over time. However, ongoing follow-up is still necessary to ensure that the patient responds correctly to treatment and spot any potential complications. Therefore, pericarditis and myocarditis require close patient health monitoring, including frequent imaging and vital sign checks [1,6,7]. The risks and benefits of intrusive procedures, such as pericardial windows, should be carefully considered. TEE should be viewed as a routine practice to reduce unnecessary surgical intervention and the danger of myocardial degeneration [5,7]. In rare cases, myocardial and pericardial inflammation can cause severe heart problems such as cardiac tamponade or sudden heart failure [7].

Anesthesiologists should be aware of the potential for pericarditis and myocarditis in patients who have received the Pfizer-BioNTech COVID-19 vaccine and take appropriate precautions to monitor and manage any potential complications during anesthesia. It is essential to closely monitor patients for any signs of heart problems during and after surgery, including heart rate or rhythm changes, chest pain, or shortness of breath. Further research is needed to better understand the long-term implications of pericarditis and myocarditis following COVID-19 vaccination with this vaccine and to develop strategies to minimize the risk of these complications during anesthesia.

Patients who have cardiac and pericardial inflammation due to the COVID-19 vaccination need careful evaluation of anesthetic options, monitoring, and precautions during surgical procedures. The anesthetic used should aim to reduce myocardial depression and arrhythmogenic potential. To minimize hemodynamic instability and myocardial depression, regional anesthetic methods such as thoracic epidural or paravertebral blocks may be preferred over general anesthesia [8,9]. If general anesthesia is required, short-acting agents such as propofol and remifentanil may be preferred over long-acting medications to reduce the duration of cardiac depression [9]. Inhaled anesthetics such as sevoflurane may also be used; however, excessive doses should be avoided to avoid hypotension and myocardial depression. Individuals with cardiac and pericardial inflammation after the COVID-19 vaccination need close hemodynamic monitoring. Invasive monitoring, such as arterial and central venous catheters, may be required to calculate cardiac output and blood pressure accurately [10]. Continuous electrocardiogram (ECG) monitoring is essential for detecting arrhythmias or ST segment changes. During the anesthetic treatment of patients with myocardial and pericardial inflammation after COVID-19 vaccination, precautions should be taken to reduce the risk of myocardial depression and arrhythmias. Avoiding hypotension and tachycardia, maintaining enough intravascular volume, and avoiding medications that may cause QT segment prolongation or arrhythmias are all precautions [11].

Furthermore, careful titration of opioids and neuromuscular blocking medicines is required to avoid respiratory depression and excessive doses that may result in cardiac depression. Conclusively, the anesthetic management of patients with cardiac and pericardial inflammation after COVID-19 vaccination requires careful consideration of anesthetic choice, monitoring, and efforts to reduce the risk of complications and provide the best possible outcomes. Consulting with a cardiologist is advised in these cases to guide anesthetic therapy and enhance patient care.

## Conclusions

In this case report, the anesthetic care for a patient with suspected COVID-19 vaccination-induced pericarditis/myocarditis during pericardial window surgery is shown. Rare but possibly catastrophic side effects of COVID-19 immunization include myocarditis and pericarditis. Anesthesia treatment for these patients requires monitoring, attention in selecting the anesthetic, and precautions to reduce the risk of complications and enhance patient outcomes. This case report highlights the need for cautious anesthetic treatment in patients having surgical operations who have suspected myocarditis or pericarditis caused by COVID-19 immunization. Furthermore, the risks and benefits of invasive therapies such as pericardial windows should be carefully considered. TEE should be regarded as a routine technique in order to minimize unnecessary surgical intervention and the risk of myocardial degeneration. To provide these patients with the best treatment possible, clinicians, educators, and researchers should be aware of these potential problems and collaborate closely with the multidisciplinary team.
